# Does medication-related osteonecrosis of the jaw affect survival of patients with Multiple Myeloma?: Exploring a large single center database using artificial intelligence

**DOI:** 10.1007/s10238-023-01100-6

**Published:** 2023-10-07

**Authors:** Max Bittrich, Regina Hetterich, Antonio G. Solimando, Markus Krebs, Sophia Loda, Sophia Danhof, Straub Anton, Xiang Zhou, Alexander Kerscher, Andreas Beilhack, K. Martin Kortüm, Leo Rasche, Hermann Einsele, Stefan Knop, Stefan Hartmann

**Affiliations:** 1 Department of Internal Medicine II, University Hospital Würzburg, 97080 Würzburg, Germany; 2 Department of Biomedical Sciences and Human Oncology, Section of Internal Medicine ‘G. Baccelli’, University of Bari Medical School Bari, 70124 Bari, Italy; 3 Comprehensive Cancer Center Mainfranken, 97080 Würzburg, Germany; 4 Department of Internal Medicine 5, Hematology and Oncology, University Hospital of Paracelsus Medical Private University, 90419 Nuremberg, Germany; 5 Department of Oral and Maxillofacial Plastic Surgery, University Hospital Würzburg, 97070 Würzburg, Germany

**Keywords:** Multiple Myeloma, Artificial intelligence, Natural language processing, Real world evidence, Propensity score matching, Osteonecrosis of the jaw, Rare diseases, Rare adverse events

## Abstract

In addition to randomized clinical trials, consideration of Real-World Evidence is necessary for mirroring clinical reality. However, processing such evidence for large numbers of patients often requires considerable time and effort. This is particularly true for rare tumor diseases such as multiple myeloma (MM) or for adverse effects that occur even more rarely. In such cases, artificial intelligence is able to efficiently detect patients with rare conditions. One of these rare adverse events, and the most discussed, following bone protective treatment in MM is medication-related osteonecrosis of the jaw (MRONJ). The association of bone protective treatment to MM outcome has been intensively studied. However, the impact of MRONJ resulting from such treatment on MM prognosis and outcome is poorly understood. In this retrospective study, we therefore investigated the long-term effects of MRONJ. We used natural language processing (NLP) to screen individual data of 2389 MM patients to find 50 out of 52 patients with MRONJ matching our inclusion criteria. To further improve data quality, we then performed propensity score matching. In comparison to MM patients without MRONJ, we found a significantly longer overall survival (median 126 vs. 86 months) despite slightly worse clinical features.

**Supplementary Information:**

The online version contains supplementary material available at 10.1007/s10238-023-01100-6.

## Introduction

Multiple myeloma (MM) accounts for 1% of all malignant tumors diagnosed worldwide [1]. Many patients face bone involvement with osteolytic lesions and bone pain. Bone protective therapies are the gold standard in MM to reduce skeletal related events (SRE) and increase bone mineralization. SREs are pathological fractures, bone marrow compression, hypercalcemia, necessity for radiotherapy or surgical treatment. In addition to calcium and vitamin D, aminobisphosphonates, mostly zoledronate, or the receptor activator NF-κ-B ligand inhibitor denosumab are used as anti-resorptive (AR) medication. AR treatment is often highly individualized among MM patients. AR drugs are shifted in a long therapy duration. The efficacy of antiresorptive drugs, such as zoledronic acid and denosumab, appears to be comparable [2].

The most discussed adverse event with bisphosphonates or denosumab is medication-related osteonecrosis of the jaw (MRONJ). This seems to be drug class side effect. The frequent use of high-dose corticosteroids in MM treatment is notable, too, as these medications have been linked to an elevated risk of MRONJ [3, 4]. While these compounds are not exclusively causing MRONJ, other related drugs such as sunitinib or bevacizumab, are not commonly used in MM treatment. Other factors also influence MRONJ occurrence [5–7], including smoking, diabetes mellitus, insufficient oral hygiene, immunosuppression and non-compliance with dental follow-up. But all of these are common and not MM-specific. According to internationally accepted standards, we defined MRONJ as jaw bone exposed for more than 8 weeks without self-healing and fistula present, history of AR medication, no craniofacial radiation in medical history and no bone metastases in the jaw [8–10]. The association between bone microenvironment and AR drugs as well as resulting osteonecrosis has been intensively studied. However, the impact on long-term progression free survival (PFS) and overall survival (OS) is not well understood.

In the available randomized clinical trials, follow-up has usually been too short for assessing possible long-term effects in OS [11]. Some long-term data demonstrate a low incidence of renal adverse events [12]. In this retrospective study, we investigated the long-term effects of MRONJ on MM patients. To improve data quality, we performed propensity score matching (PSM) with a clinically matched control group.

## Methods

### Patients

We conducted a retrospective single-center study. Inclusion criteria were age ≥ 18 years, histologically confirmed MM, primary MM diagnosis between 1993 and 2017, previous AR treatment, fully available medical history in an electronic health record system and available details on medication and dosing. Patients with at least one of the following aberrations were considered as high-risk: deletion (17p), translocation (4; 14), translocation (14; 16), translocation (14; 20) and gain (1q) (with at least four copies). All information was obtained before data cut-off date March 31, 2019.

### Natural language processing (NLP) and propensity score matching (PSM)

The entire data collection process was supported by the artificial intelligence (AI) ARIES. This AI process is described elsewhere [13]. In order to compare, we selected an equal sized control (CTRL) group based on the inclusion criteria and PSM. PSM variables were sex, year of primary diagnosis, age at primary diagnosis, cytogenetic risk assessment, international staging system stadium (ISS), revised ISS stadium (R-ISS), MM heavy chain involvement, and MM light chain subtype.

### Response, MRONJ, ethical approval

The tumor stage was assigned according to the revised international staging system (R-ISS). We calculated OS, time to first subsequent therapy (TFST), progression free survival (PFS1), PFS from primary diagnosis to second relapse (PFS2), overall response (ORR) and duration of response (DOR). The median overall survival was defined as the time from the date of initial diagnosis of MM to the patient's death or the censored last time point. Treatment response was assessed according to the international myeloma working group criteria [14, 15]. Cases that occurred before the initial MRONJ description in 2003 [10] were included according to clinical images, symptoms and retrospective assessment by a cranio-maxillo-facial surgeon. MRONJ severity was assessed in line with the AAOMS recommendations [8]. Our study was authorized by the institutional review board of the University of Würzburg (reference number 20200129 02).

### Statistics

Descriptive data were reported using absolute and relative frequencies. Median and mean values were listed with the minimum and maximum values. We used student’s t-test for parametric or Mann–Whitney *U* test for non-parametric values to examine significant differences. With categorial variables we used fisher’s exact test where possible. When there were more than two categories present, we used Chi-square test. For correlation analysis we applied Spearman method for continuous variables and logistic regression to correlate categorial with continuous variables. Survival statistics were performed according to Kaplan–Meier and compared by Gehan–Breslow–Wilcoxon test for better results in early time points. The hazard ratio (HR) was quantified with the Mantel–Haenszel method. Multivariate analysis was done via Cox proportional hazard regression. Data were equally weighted. All tests were two-tailed where feasible. These analyses were performed with Graph Pad Prism for Windows (Version 9.4.1, San Diego, CA, USA). The significance level for rejecting the null hypothesis was set to *p*-value < 0.05. The confidence level for the confidence interval (CI) was set at 95%.

## Results

First, we surveyed patient characteristics and known MM prognostic parameters to identify potential differences between patients with and without MRONJ. Searching our real-world single-center cohort of 2389 MM patients, we identified 52 patients with MRONJ. 50 from these 52 patients met the inclusion criteria. Notably, 16 of these 52 patients (31%) began AR therapy before on January 1, 2005, when standardized dental evaluations were implemented at our center. And eleven of these 52 (21%) received MRONJ diagnoses before the condition's initial description in 2003. Osteonecrosis of the jaw cases before 2003 were included based on a cranio-maxillofacial surgeon's statement. Of note, among all 2,389 patients, 878 already started AR treatment before January 1, 2005. Subsequent PSM revealed 50 corresponding MM patients without MRONJ. These patients served as the control. We propensity score-matched important prognosis values. The differences in sex, age at primary diagnosis, cytogenetic risk assessment, ISS, revised ISS stadium (R-ISS), MM heavy chain involvement and MM light chain subtype were not significant. Statistically, patients from the MRONJ group received MM diagnosis 5.5 years earlier than patients belonging to the CTRL group (*p* < 0.001; CI 3.408 to 7.592). The patient characteristics are summarized in Table 1.

**Table 1:** Patients’ characteristics

	MRONJ	CTRL		Total
	*n* (Range)	*n* (Range)	*p*-Value	*n* (Range)
Age	64 (40–82)	66 (42–86)	0.535	66 (40–86)
LOT	4 (1–12)	3 (1–8)	0.087	3 (1–12)
Year Diagnosis	2007 (1993–2017)	2012 (2001–2017)	< 0.001	2009 (1993–2017)

		*n* (%)	*n* (%)		*n* (%)
Sex	Male	35 (70)	38 (76)	0.653	73 (73)
	Female	15 (30)	12 (24)		27 (27)
MM subtype	Ig G	28 (56)	32 (64)	0.513	60 (60)
	Ig A	15 (30)	10 (20)		25 (25)
	LC	7 (14)	8 (16)		15 (15)
Light chain type	Kappa	36 (72)	39 (78)	0.645	75 (75)
	Lambda	14 (28)	11 (22)		25 (25)
ISS	I	20 (40)	18 (36)	0.878	38 (38)
	II	18 (36)	18 (36)		36 (36)
	III	12 (24)	14 (28)		26 (26)
R-ISS	I	12 (24)	10 (20)	0.826	22 (22)
	II	29 (58)	29 (58)		58 (58)
	III	9 (18)	11 (22)		20 (20)
Cytogenetic stratification	Standard risk	36 (72)	35 (70)	> 0.999	71 (71)
	High risk	14 (28)	15 (30)		29 (29)
Cytogenetic high risk aberrations in detail (more than one possible)	del (17p)	5 (10)	9 (18)	0.388	14 (14)
	*t* (4;14)	5 (10)	7 (14)	0.760	12 (12)
	*t* (14;16)	1 (2)	1 (2)	> 0.999	2 (2)
	*t* (14;20)	0 (0)	0 (0)	> 0.999	0 (0)
	gain (1q21) (≥ 4 cps)	11 (22)	8 (16)	0.611	19 (19)

*CTRL* Propensity score-matched control group, *cps* copies, *IgA* immunoglobulin A, *IgG* immunoglobulin G, *ISS* international staging system, *LC* Light chain myeloma, *LOT* Line of therapy, *MM* multiple myeloma, *MRONJ* medication associated osteonecrosis of the jaw group, *R-ISS* revised international staging system

Overall, the majority of patients were male (73%). The median age in the entire patient population was 66 years (40–86). Patients in the MRONJ group were on average 2 years younger than those in the CTRL group, which was not significant (*p* = 0.535). The Eastern-Cooperative Oncology Group score was not matched, but averaged one in median in both groups. The difference was not significant (*p* = 0.577). Co-morbidities (smoking, drinking, obesity, cardiovascular diseases) were not matched. The MRONJ group tended to be more frequently affected than the CTRL group (82% vs. 66%). But the difference was not significant (*p* = 0.101). Secondary malignancies were more frequent present in the MRONJ group (20% vs. 16%), which was not significant (*p* = 0.795). Of MM progression parameters, Bence Jones proteinuria was equally present in both groups. In the MRONJ group, less patients had impaired renal function, compared with the CTRL group, but this was not significantly different (40% vs. 56%, *p* = 0.660). All other prognosis parameters – e.g., bone marrow infiltration (47% vs. 49%, *p* = 0.726), CAST nephropathy (2% vs. 4%, *p* > 0.999) showed no significant difference. Details are given in Table 2 and the supplement. In short, both patient groups were approximately similar in main MM details besides the year of diagnosis.

**Table 2:** MM prognosis parameters

		MRONJ	CTRL		Total
		*n* (%)	*n* (%)	*p*-value	*n* (%)
ECOG	0	18 (36)	19 (38)	0.577	37 (37)
	1	26 (52)	28 (54)		54 (54)
	2	6 (12)	3 (6)		9 (9)
Extramedullary disease	Yes	5 (10)	9 (18)	0.388	14 (14)
	No	45 (90)	41 (82)		86 (86)
Hypercalcemia	Yes	6 (12)	4 (8)	0.741	10 (10)
	No	44 (88)	46 (92)		90 (90)
Renal function impairment	Yes	13 (26)	15 (30)	0.824	28 (28)
	No	37 (74)	35 (70)		72 (72)
CAST nephropathy	Yes	1 (2%)	2 (4%)	> 0.999	3 (3)
	No	49 (98)	48 (96)		97 (97)
Anemia	Yes	25 (50)	32 (64)	0.225	57 (57)
	No	25 (50)	18 (36)		43 (43)
Present bone lesions	Yes	41 (82)	44 (88)	0.577	85 (85)
	No	9 (18)	6 (12)		15 (30)
Smoking, drinking, obesity, cardiovascular diseases	Yes	41 (82)	33 (66)	0.110	74 (74)
	No	9 (18)	17 (34)		26 (26)
Secondary malignancies	Yes	10 (20)	8 (16)	0.795	18 (18)
	No	40 (80)	42 (82)		82 (82)

*CAST* Urinary light chain formation of plugs, *ECOG* eastern-cooperative oncology group score

We then analyzed bone involvement and AR treatment in more detail. There were similar proportions of SRE in both groups (60% vs. 62%, *p* > 0.999). There were similar pathological fractures in both groups (38% vs. 42%, *p* = 0.838). Hypercalcemia (12% vs. 8%, *p* = 0.741), bone marrow compression (12% vs. 18%, *p* = 0.577) and surgical treatment or irradiation of the bone (40% vs. 46%, *p* = 0.686) occurred to similar degrees in both groups. None of the differences were statistically significant. Details are highlighted in Table 3.

**Table 3:** SRE

		MRONJ	CTRL		Total
		*n* (%)	*n* (%)	*p*-value	*n* (%)
SRE	Yes	30 (60)	31 (62)	> 0.999	61 (61)
	No	20 (40)	19 (38)		39 (39)
Pathological fractures	Yes	17 (38)	21 (42)	0.838	38 (38)
	No	33 (66)	29 (58)		62 (62)
Hypercalcemia	Yes	6 (12)	4 (8)	0.741	10 (10)
	No	44 (88)	46 (92)		90 (90)
Bone marrow compression	Yes	6 (12)	9 (18)	0.577	15 (15)
	No	44 (88)	41 (82)		85 (85)
Surgical treatment or irradiation of the bone	Yes	20 (40)	23 (46)	0.686	43 (86)
	No	30 (60)	27 (54)		57 (57)

*SRE* Skeletal related events

Both groups received AR therapy. Sixteen out of the 50 MRONJ patients (32%) and four out of the 50 CTRL patients (8%) started AR treatment at our center before January 1, 2005. All patients could have more than one AR drug and undergo more than one treatment interval. In both groups, AR therapy was initiated 61 times each. In both groups, there were patients who were sequentially treated with more than one AR in the course of their illness. Use of AR drugs was evenly distributed between the two groups (*p* = 0.129). The drugs in both groups were zoledronic acid (62% vs. 50%, *p* = 0.574), pamidronic acid (28% vs. 20%, *p* = 0.489) and denosumab or others e.g., clodronic acid, ibandronic acid or alendronic acid (20% vs. 38%, *p* = 0.129). A total of 36 times zoledronic acid was started in both the MRONJ and CTRL groups (72% vs. 72%, *p* > 0.999). In the MRONJ group, Pamidronate was also administered sequentially in 7 cases, either before or after. In the CTRL group, there were 6 cases sequentially started with Pamidronate. In an additional 3 cases in the CTRL group, a switch to another AR drug was made.

AR treatment intervals were not evenly distributed. The most frequent treatment intervals for MRONJ versus CTRL group were 4 weeks (90% vs. 66%, *p* = 0.004) in comparison to 8 weeks (2% vs. 6%, *p* = 0.618) or 12 or more weeks (8% vs. 30%, *p* = 0.005). Patients in the MRONJ group had received AR therapy more often in 4-week intervals than the CTRL group. In the MRONJ group, zoledronate or pamidronate was initiated a total of 53 times. Some patients were treated with zoledronate and pamidronate sequentially and at varying intervals. In 49 cases, treatment began at a 4-week interval with zoledronate (*n* = 33) or pamidronate (*n* = 16); in no case was a 6-week interval used, while 3 cases had a 12-week interval (zoledronate: *n* = 2, pamidronate: *n* = 1), and one case had a 24-week interval with zoledronate. The majority of cases in the MRONJ group were treated at the shortest possible interval of 4 weeks with ARs. The number of patients receiving a different AR was too small to determine meaningful differences regarding the interval. The CTRL group received AR therapy more often in 12-week intervals. In the CTRL group, zoledronate or pamidronate was initiated 52 times. In the CTRL group, 36 cases were treated at a 4-week interval with zoledronate (*n* = 24) or pamidronate (*n* = 12); 4 cases had a 6-week interval with zoledronate; 11 cases had a 12-week interval (zoledronate: *n* = 7, pamidronate: *n* = 4); and one case had a 24-week interval with zoledronate. The number of patients receiving a different AR was too small to determine meaningful differences regarding the interval.

In both the MRONJ group and the CTRL group, a total of 61 AR therapies were initiated. In the MRONJ group, 60 of these therapies were discontinued or interrupted, with MRONJ being the reason in all cases. In the CTRL group, AR therapy was interrupted or discontinued 16 times until data collection ended. The reasons were adverse side effects in 4 cases, patient death in 8 cases, and a planned dental extraction in 4 cases. Of the 50 patients who experienced MRONJ, 11 (22%) received AR therapy again following this event. Four of these 11 patients developed a second MRONJ, which represented 36% of patients who had received repeated AR therapy.

**Fig. 1:**
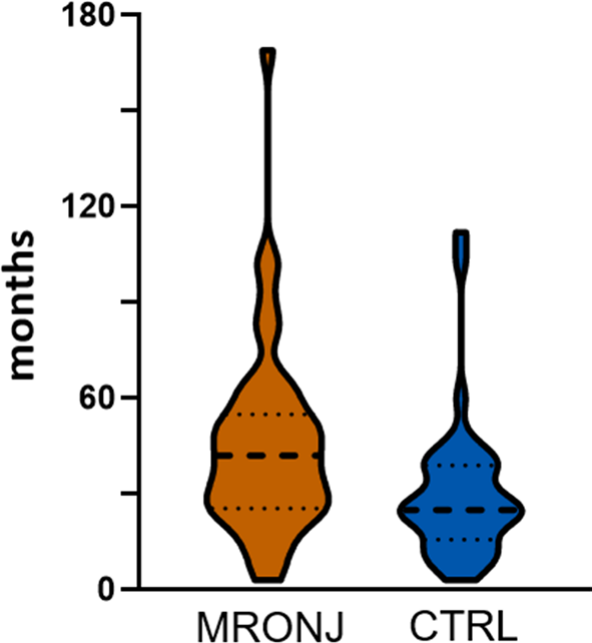
Duration of AR treatment (MRONJ vs. CTRL group)

The duration of AR therapy was also not evenly distributed. On average, patients in the MRONJ group were treated with ARs for 45 (CI: 37–53) months and patients in the CTRL group for 29 (CI: 22–36) months. AR therapy lasting a maximum of 24 months was observed in 10 patients (20%) in the MRONJ group, while a significantly higher number of 20 patients (40%) in the CTRL group had the same duration (*p* = 0.049). The AR therapy lasted 25–48 months for 20 MRONJ patients (40%) versus 18 CTRL patients (36%, *p* = 0.837), 49 to 72 months for 14 MRONJ patients (28%) versus one CTRL patient (2%, *p* < 0.001), and longer than 72 months for 6 MRONJ patients (12%) versus 2 CTRL patients (4%, *p* = 0.269). For one MRONJ patient (2%) versus nine CTRL patients (18%), the exact duration of AR therapy was unknown (*p* = 0.016). Patients in the MRONJ group received AR therapy in total for longer on average than the CTRL group. This difference was significant (*p* = 0.006). For the cut-off of AR therapy for more than 24 months, the difference was also significant (80% vs. 42%, *p* < 0.001). Details are highlighted in Fig. 1.

The duration of AR therapy was determined for each patient in therapy months. The bisphosphonate therapy lasted a maximum of 24 months for 10 patients (20%) in the MRONJ group and for 20 patients (40%) in the CTRL group. It lasted 25–48 months for 20 MRONJ patients (40%) vs. 18 CTRL patients (36%), 49–72 months for 14 MRONJ patients (28%) vs. one CTRL patient (2%), more than 72 months for 6 MRONJ patients (12%) vs. 2 CTRL patients (4%). In one vs. 9 cases, the length of therapy was unknown. For the cut-off of 24 months, the difference was significant (*p* < 0.001).

For the period from 1993 to 2017, NLP identified 52 MM patients suffering from MRONJ—representing an MRONJ incidence rate of 2.17%. Regarding localization and severity of MRONJ, we saw the mandible affected four times in stage 1 (8%), 18 times in stage 2 (36%) and three times in stage 3 (6%). The maxilla was affected twice in stage 1 (4%), nine times in stage 2 (18%) and three times in stage 3 (6%). Both jaws were simultaneously affected once in stage 1 (2%) and once in stage 2 (2%). In 9 cases (18%), MRONJ location was unknown due to missing documentation or images. Overall, the mandible was more frequently affected than the maxilla, and in stage 3, the two jaws were equally affected regarding frequency. Of the 50 patients who experienced MRONJ, 11 (22%) received AR therapy again following the MRONJ event. Four of these 11 (36%) patients subsequently developed a second MRONJ. The treatment procedure was conservative in 20% of patients and surgical in 60%. In 20%, the procedure was unknown.

**Fig. 2:**
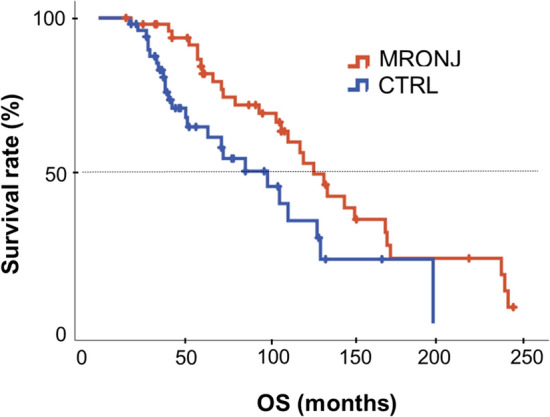
OS (MRONJ vs. CTRL)

Next, we investigated the impact of MRONJ on PFS and OS. The follow-up in this study was 83 (CI: 72–94) months. Follow-up duration was significant longer in the MRONJ group than in the CTRL group (104 vs. 63 months, *p* < 0.001). The median PFS1 was 35 (CI: 34–56) months in the MRONJ group and 27 (CI: 27–43) months in the CTRL group. This difference was not significant (*p* = 0.165). The median PFS2 was 60 (CI: 55–76) months in the MRONJ group and 42 (CI: 39–65) months in the CTRL group. This difference was not significant either (*p* = 0.096). The median OS was 111 months for all 100 patients. Patients in the MRONJ group had a median OS of 126 months. In the CTRL group, it was 86 months. The ratio of the median survivals was 1.47 (CI: 0.862–2.49). The median OS in the MRONJ group was significantly longer than that in the CTRL group (*p* = 0.009). The median OS difference between the groups was 40 months. We assessed the survival benefit statistically associated with a MRONJ diagnosis. The HR for OS was 0.494 (CI: 0.273–0.895) of the MRONJ group in comparison to CTRL (Fig. 2).

OS in the MRONJ and CTRL group were calculated according to Kaplan–Meier. The patients of the MRONJ group reached a median OS of 126 months. The CTRL patients had a median OS of 86 months. The median OS of the patients of the MRONJ group was 40 months higher than that of the CTRL group (*p* = 0.009). The quantified benefit was a HR 0.494 (CI: 0.273–0.895) in favor of the MRONJ group.

In Germany, AR therapy for MM is usually administered for at least 24 months. The period is usually extended, if there are no negative effects, with the treatment interval increased to 3 months. Patients who had AR therapy for more than 24 months survived for longer than those whose therapy was 24 months or less. In the subgroup (*n* = 70) with 24 months or more of AR therapy, the median OS was 134 months. The difference between MRONJ and CTRL in this subgroup was not significant (132 vs. 196 months, *p* = 0.760). In the smaller subgroup (*n* = 30) with AR therapy of shorter than 24 months duration, the median OS was 64 months. The difference between MRONJ and CTRL in this subgroup was not significant either (67 vs. 52 months, *p* = 0.343). The differences between 24 months or more AR therapy versus less than 24 months of AR therapy subgroup was significant (*p* < 0.001).

In the next step, we tried to identify confounding parameters. One obvious confounder was the year of diagnosis. Year of diagnosis could not be fully matched in PSM, because there were no nearest neighbors with a better suiting despite 2,389 MM patients’ data. On average, patients from the MRONJ group did receive MM diagnosis 5.5 years earlier in 2007 (1993–2017) than patients from the CTRL group in 2012 (2001–2017). In this respect, it is possible that the treatment regime has a key role. MM treatment regimes were not included in PSM, because only a priori information should be used in PSM, and plasma cell depleting therapies are too diverse to be matched.

In both groups, therapy started promptly after diagnosis. Time to first treatment was one month in both groups (*p* = 0.785). The median number of therapy lines in the MRONJ group was 4 (1–12). The median in the CTRL group was 3 (1–8) lines (*p* = 0.086). The modern, high efficiency drugs proteasome inhibitors (PIs) and immunomodulatory drugs (IMiDs) tended to be less used in the MRONJ group than in the CTRL group. With 62% vs. 86% (*p* = 0.011) for PIs, proportions were significantly different. For IMiDs, the proportions were 66% vs. 78% (*p* = 0.265). Some patients received both substance classes. The proportions were 48% vs. 72%. This difference was significant (*p* = 0.024). CD38-targeting antibodies were not in use in the time horizon (1993–2017) of our study. High-dose therapy with autologous transplantation (autoSCT) was less frequent in the MRONJ group compared to CTRL (54% vs. 64%, *p* = 0.416). This was also true for primary tandem transplantation (32% vs. 50%, *p* = 0.103). Single transplant concept (16% vs. 10%, *p* = 0.554) and delayed second transplantation concept at relapse (6% vs. 4%, *p* ≥ 0.999) were not significantly different. A third transplantation at relapse occurred in both groups with the same frequency (10% vs. 10%, *p* > 0.999). There was a tendency towards fewer allogeneic transplantations performed in the MRONJ group (6% vs. 14%, *p* = 0.318). Early relapse within 18 months after autologous transplantation occurred in 20% vs. 30% (*p* = 0.356). Overall, the CTRL group tended to undergo autologous transplantation more frequently and received more high-dose therapies and transplants. The differences were not significant. Details are given in the supplement.

Seven patients (14%) became PI-refractory in the MRONJ group and 15 patients (30%) in the CTRL group (*p* = 0.090). Regarding IMiDs, fourteen patients (28%) in the MRONJ group became refractory vs. 21 patients (42%) in the CTRL group (*p* = 0.208). Thus, both groups were similarly affected by refractory disease. Details are shown in Table 4.

**Table 4:** MM therapies

		MRONJ	CTRL		Total
		*n* (%)	*n* (%)	*p*-value	*n* (%)
PI treated	Yes	31 (62)	43 (86)	0.011	74 (74)
	No	19 (38)	7 (14)		26 (26)
IMiD treated	Yes	33 (66)	39 (78)	0.265	72 (72)
	No	17 (34)	11 (22)		28 (28)
PI and IMiD treated	Yes	24 (48)	36 (72)	0.024	60 (60)
	No	26 (52)	14 (28)		40 (40)
PI refractory	Yes	7 (14)	15 (30)	0.090	22 (22)
	No	43 (86)	35 (70)		78 (78)
IMiD refractory	Yes	14 (28)	21 (42)	0.208	35 (35)
	No	36 (72)	29 (58)		65 (65)
AutoSCT total	Yes	27 (54)	32 (64)	0.416	59 (59)
	No	23 (46)	18 (36)		41 (41)
Primary Tandem autoSCT	Yes	16 (32)	25 (50)	0.103	41 (41)
	No	34 (68)	25 (50)		59 (59)
Delayed tandem autoSCT	Yes	3 (6)	2 (4)	> 0.999	5 (5)
	No	47 (94)	48 (96)		95 (5)
Single autoSCT	Yes	8 (16)	5 (10)	0.554	13 (13)
	No	42 (84)	45 (90)		87 (87)
Early relapse autoSCT	Yes	10 (20)	15 (30)	0.356	25 (25)
	No	40 (80)	35 (70)		75 (75)
Third autoSCT	Yes	5 (10)	5 (10)	> 0.999	10 (10)
	No	45 (90)	45 (90)		90 (90)
AlloSCT	Yes	3 (6)	7 (14)	0.318	10 (10)
	No	47 (94)	43 (86)		90 (90)

*autoSCT* High-dose therapy and autologous transplantation, *alloSCT* High-dose therapy and allogeneic transplantation, *IMiD*  immunmodulatory drugs, *PI* proteasome inhibitors

**Fig. 3:**
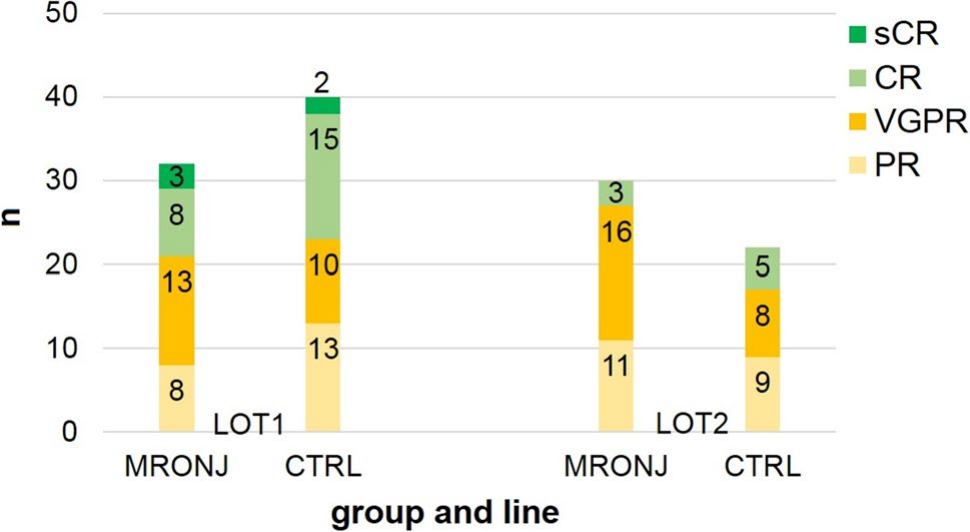
Therapy response to first and second line of therapy (MRONJ vs. CTRL)

The MRONJ group achieved an ORR of 64% in LOT1, 8 patients in the MRONJ group achieved partial remission (PR), 13 patients achieved very good PR (VGPR), and 11 patients achieved complete remission (CR) or better. In the CTRL group, the ORR in LOT1 was 80%. 13 patients achieved a PR, 10 VGPR, and 17 CR. The difference was not significant (*p* = 0.367). In LOT2, the ORR was 72% in the MRONJ group and 56% in the CTRL group (*p* = 0.335). The MRONJ group had a lower ORR in LOT1 and a higher ORR in LOT2 than the CTRL group (LOT1 68% vs. 80%; LOT2 72% vs. 56%). Details are shown in Fig. 3.

In both groups, ORR was higher in LOT1 than in LOT2. In LOT1, three patients in the MRONJ group and two patients in the CTRL group reached stringent CR (sCR). For CR, the equivalent figures were eight and 15, for VGPR 13 and 10, and for PR eight and 13. In LOT2, the proportions were reversed: no patient in either of the groups reached sCR, for CR the figures were three (MRONJ) and five (CTRL), for VGPR 16 and eight, and for PR 11 and 9. None of the differences were significant.

The overall DOR in LOT1 was 31 (CI: 24–39) months. The DOR for MRONJ vs. CTRL group was 36 vs. 28 months (*p* = 0.273). The overall DOR in LOT2 was 16 (CI: 11–21) months. The DOR in LOT2 for MRONJ vs. CTRL group was 19 vs. 13 months (*p* = 0.231). The difference in overall DOR between LOT1 and LOT2 was significant (*p* = 0.002). In correlation analysis, DOR in LOT1 and LOT2 correlated significantly with the duration of AR therapy (in LOT1: *p* < 0.001, *r* = 0.378; in LOT2: *p* = 0.021, *r* = 0.303).

To search for further confounders, we calculated HRs in multivariate analysis. With HR 20.7 (*p* = 0.033), the occurrence of secondary malignancies significantly impacted estimated survival rates. Secondary malignancies were observed in both groups: 10 patients (20%) in the MRONJ group and 8 patients (16%, *p* = 0.795) in the CTRL group experienced secondary malignancies. The types of secondary malignancies included acute myeloid leukemia, prostate cancer, colorectal cancer, breast cancer, pancreatic cancer, and gastric cancer. Additionally, the absence of bone involvement tended to be a negative predictor of survival in our cohort (HR 32.6, *p* = 0.070). ECOG performance score showed a disadvantage in higher levels (ECOG 1: HR 3.9, ECOG 2 HR 17.9). This was not significant (ECOG 1: *p* = 0.410, ECOG 2: *p* = 0.279). Impairment of renal function (HR: 3.88, *p* = 0.245) and anemia (HR: 3.44, *p* = 0.291) had also negative impact in tendency. Regarding MM therapies, patients who had not undergone autoSCT tended to have a worse survival (HR 3.4, *p* = 0.313). In addition, if patients did not receive modern drugs, the estimated survival was potentially worse (HR 2.7, *p* = 0.526). A positive effect on estimated survival tended to be the absence of cardiovascular risk factors (HR: 0.42, *p* = 0.420). Details are shown in Table 5.

**Table 5:** Multivariate analysis

		Estimated hazard ratio	CI	*p*-value
Extragradient	g/l	0.99	0.94–1.03	0.498
Bence Jones proteinuria	mg/l	1.00	1.00–1.00	0.171
β2-microglobulin	mg/l	1.26	0.93–1.81	0.150
LDH	U/l	0.98	0.95–1.00	0.083
Bone marrow infiltration	%	1.05	0.98–1.13	0.183
ECOG	1	3.80	0.19–187	0.410
	2	17.90	0.08–6468	0.279
Present bone lesions	No	32.60	0.55–1733	0.070
Smoking, drinking, obesity, cardiovascular diseases	No	0.42	0.05–4.38	0.420
Secondary malignancies	Yes	20.70	2.05–824	0.033
Renal function impairment	Yes	3.88	0.36–52.50	0.245
Anemia	Yes	3.44	0.36–45.20	0.291
SRE	Yes	0.88	0.07–7.90	0.905
PI or IMID treated	No	2.70	0.08–66.90	0.526
AutoSCT	No	3.40	0.29–44.4	0.313

*autoSCT* High-dose therapy melphalan and autologous transplantation, *ECOG* eastern-cooperative oncology group, *IMiD* immunmodulatory drugs, *LDH* lactate dehydrogenase, *PI* proteasome inhibitors, *SRE* skeletal related events

To address the results of the multivariate analysis, we removed all the patients with secondary malignancies from the OS calculation. However, the survival benefit of the MRONJ group was still significant. The MRONJ group without secondary malignancies now had a median survival of 132 months versus 99 months in CTRL group (ratio 1.33, CI of ratio: 0.732 to 2.43, *p* = 0.015). The quantified Hazard ratio was comparable with 0.427 (CI: 0.214–0.852) in comparison to 0.494 (CI: 0.273–0.895) before. In conclusion, secondary malignancies were one important confounder, but the impact of MRONJ on survival remained.

## Discussion

To the best of our knowledge, this is the first work of this scale investigating the influence of MRONJ on the outcome of MM patients. Moreover, this is the first study using an AI system to address clinical research in MM. NLP helped us to analyze a large data set of MM patients (*n* = 2389) to assess rare adverse events in a real-world evidence setting. There is yet no published data on NLP in MM. However, it is possible to use NLP to address MM diagnosis in serum electrophoresis [16]. Furthermore, NLP is used in clinical research of pathology reports, breast cancer and colorectal carcinoma [17–19].

Our most significant finding was that patients in the MRONJ group had a considerably longer survival time than those in the CTRL group (126 vs. 86 months). This result is interesting, as other most recent data from Hata et al. also suggest that MRONJ diagnosis may be statistically associated with improved survival in various tumor diseases, including MM. In Hata et al.'s study, patients without MRONJ had a median survival of 18 months, while patients with MRONJ lived for 91 months. Conversely, Lu et al. reported a significantly worse outcome for MM patients with MRONJ [20]. Focusing on MM Hata et al. observed a survival time of 41 months in 86 zoledronic acid-treated MM patients [21], which is considerably shorter than our observed median survival time of 111 months for all 100 analyzed MM patients. Of note, only seven out of these 86 patients developed MRONJ in Hata’s single-center retrospective study [22]. Cabras et al. have also shown a shorter survival time of 31 months in MM patients after MRONJ onset [23]. Recently, Pulte et al. have presented data demonstrating an improvement in survival times for MM patients. Their study revealed a nearly twofold increase in the relative survival times between 2002–2006 and 2012–2016, further supporting the evolving understanding of survival trends in this patient population [24]. This might potentially account for the extended overall survival we identified in our study. Moreover, Fusco et al. recently documented a long median survival time of 77 months in MM patients receiving AR treatment [25]. Although there is other evidence indicating a possibly prolonged survival time, our study adds valuable insight and further expands the existing knowledge in MRONJ an MM.

We tried to find any potential cause or origin of this statistical association with OS. Important MM variables were taken into account by using PSM. These variables included sex, year of primary diagnosis, age at primary diagnosis, cytogenetic risk assessment, revised ISS stadium (R-ISS), MM subtype and light chain type. SRE showed equal frequency in both groups. The year of primary diagnosis could not be equally matched in PSM, because there were no nearest neighbors with a better suiting despite our larger data set. Thus, it seemed to be a minor confounder, because better therapies and better supportive treatment were available in later years. However, the CTRL group with worse OS (not the MRONJ group) was diagnosed 5.5 years later. Thus, this should not contribute to a survival benefit for the MRONJ group. In the multivariate analysis, year of diagnosis variable could not be added in the calculation, because there was a collinearity with OS.

Besides year of diagnosis, secondary malignancies had significant influence on survival. Patients with secondary malignancies did have worse predicted survival. But even excluding these patients from OS calculation did not alter the survival benefit of the MRONJ group substantially.

The general condition, hypercalcemia, anemia, bone involvement and soft tissue involvement all did not differ significantly in both groups. And the multivariate analysis showed no significant differences on OS.

However, a higher ECOG performance score appeared to have a negative impact. The MRONJ group seemed to be more adversely affected by higher ECOG scores (see Table 2). If applicable, this should lead to poorer survival outcomes for the MRONJ group.

Renal Function was slightly better in the MRONJ group. However, the differences were small, so they cannot be considered the main reason for the significantly longer survival of the MRONJ group. Nevertheless, we point out here that renal insufficiency was more prevalent among the patients in our study than in general. Proportions are more usually less than 20% such as 19% [26] and 17% [27]. It is possible, however, that our high values arise from different definitions of renal insufficiency. We used the new, and current, KDIGO classification system for renal insufficiency. The estimated hazard on survival was also higher for patients with impairment of renal function. While renal adverse events seems to low in numbers under ongoing AR treatment [12].

Cardiovascular concomitant disease in general reduces survival even in well-treated cancer [28]. In our study there were more co-morbidities in the MRONJ group. This makes our finding of longer OS in the MRONJ group even more remarkable. However, we recognize that not including co-morbidities in PSM is a limitation of this analysis, as it could potentially have reduced bias and further minimized the influence of confounding factors in our study. The MRONJ group was also worse in other prognostic characteristics like autoSCT status and therapy regimes with better drugs. The slightly divergent baseline between the groups in terms of tumor stage and CRAB criteria may be a minor cause for the significantly longer OS of the MRONJ group. The prevalence of anemia was not significantly different between groups- and was less at diagnosis among MM patients than the 75% reported elsewhere [29]. Bone involvement was also not significantly divergent between the two groups. However, bone involvement in our two groups (82 and 88%) was may be higher than the range of 67% [26] to 88% [29] in the previous literature. Bone involvement is also quantified by SREs, and these were about 60% initial diagnosis in our study and similar between the two groups. Another study [30] reported prevalence of 42%. However, in contrast to our study, the authors did not include hypercalcemia as an SRE. In combination, bone involvement is unlikely to explain longer OS in the MRONJ compared to the CTRL group in our study. Bone involvement did not differ markedly between our groups or with literature values.

Extramedullary disease is an important parameter and worsens the prognosis and therapy response in MM. The MRONJ group had comparable soft tissue involvement with adjacent or non-adjacent involvement to the bone (10% vs. 18%). In total, therefore, we do not consider that the different degree of soft tissue involvement determines the longer OS of the MRONJ group.

The response to therapy is probably not directly determined by MRONJ or bone supportive therapy but was slightly different in the MRONJ patients compared to CTRL patients. The MRONJ group received more pretreatment with on average one LOT more. This should result in a disadvantage for the MRONJ group. New and more efficacious agents like PI and IMiDs were used more frequently in the CTRL group, which should also result in a disadvantage for the MRONJ group. Furthermore, significantly fewer patients in the MRONJ group were treated with prognostically favorable autoSCT [19, 31] compared to the CTRL group. This is most likely related to the earlier initial diagnosis date of the MRONJ group and the associated therapeutic concepts. ORR in LOT1 and LOT2 was not evenly distributed between the groups. In LOT1, the ORR was lower in the MRONJ group than in the CTRL group. In LOT2, however, the ORR was higher in the MRONJ group than in the CTRL group. None of the MM progression parameters were significantly different between the MRONJ and the CTRL group at the time of initial diagnosis. PSM ensured a homogeneous distribution. Therefore, the reason for the different ORR in LOT1 cannot be found in different biological baseline conditions. The ORR was most likely lower in LOT1 in the MRONJ group due to the earlier diagnosis of the MRONJ group by 6 years over time. This should result in a worse therapy outcome due to less therapy efficacy without modern substances. In contrast, for the MRONJ group, ORR in LOT2 was higher than in the CTRL group. It is reasonable to assume that the inverse ratio in LOT2 is due to the new and more recently approved agents used. There was no significant difference in the distribution of PI or IMiD based therapy regimens, AR preparations and in the mode of application of AR therapy between the groups.

High-dose corticosteroids, which have been associated with an increased risk of MRONJ, are also commonly utilized in the treatment of MM. This relationship underscores the potential complexities and challenges involved in managing patients with this condition while ensuring the most effective treatment approach [7, 9].

Besides MM therapy, we observed significant differences in the duration of AR therapy and in the administration interval as well as in the resulting cumulative total dose. The longer the patients lived, the longer the AR therapy was given. It is therefore reasonable to assume that the longer OS could be associated with these therapy factors. It is plausible that the extended overall survival observed may be attributed to the benefits of the AR therapeutic approach. The mean duration of AR therapy until the development of MRONJ was 45 months in our study. This value is slightly higher than the 39 [32], 20 months [7] and 33 [33] months reported in the literature. A possible explanation for this discrepancy is the year of MM diagnosis. The MRONJ group patients were diagnosed earlier (1993–2017) than the CTRL group. However, the MRONJ was only scientifically described in 2003 [10]. But ONJ without AR association was already known for many years. It is worth noting that our center has implemented treatment guidelines for AR and potential ONJ since 2005. The few earlier cases (20) were included regarding a retrospective assessment of available images and clinical courses by a cranio-maxillo-facial surgeon. On the one hand, the longer and more frequently administered AR therapy in the same period of time produces a longer OS. On the other hand, the incidence of MRONJ has been found to rise with the duration of exposure to AR therapy, indicating a potential correlation between treatment length and the development of this adverse effect [8, 34, 35]. In our study, MRONJ affected the mandible twice as frequently as it affected the maxilla. This fits the general picture as the mandible is more frequently affected by MRONJ [8, 36, 37]. The distribution of patients between severity classes in our study (stage 1 18%, stage 2 68%, stage 3 14%) was similar to the distribution reported previously (22, 55, and 11%) [37].

It is noteworthy that SREs, as demonstrated in our findings, occurred in both the MRONJ group and the CTRL group during the course of the disease, despite AR treatment. This questions the common use of AR in MM regarding efficacy versus safety concerns. The long duration of AR therapy does not seem to have an influence on the reduction of SREs, although individual bone status was not taken into account. However, the reduction in quality of life associated with MRONJ is crucial. Two questions result from this. The first is at what point does AR therapy no longer provide a significant benefit to overall survival (OS). And the second is, at that point, does AR therapy still increase the likelihood of MRONJ and reduce quality of life. Unfortunately, we cannot answer these questions with the data from our study. However, there is now consensus that ARs should at least be considered in all patients regardless of osteolysis [30]. At our center, we administering AR therapy for MM patients over a 24-month period, with monthly treatments. After the initial period, individualized extensions may be considered on a quarterly basis, depending on the patient's specific needs, the risk and response to the therapy. Taking into account the potential occurrence of MRONJ and our findings on survival and outcomes, it appears reasonable to prolong the treatment beyond 24 months, particularly for patients who demonstrate a favorable response to AR therapy. For certain patients, the discontinuation of AR therapy should also be taken into consideration [38]. In the future, it may be feasible to identify genetic markers or surrogate parameters which can effectively distinguish a favorable subgroup in AR therapy.

In our work, MRONJ incidence was 2.17% over a period of 26 years. More recently, incidences have tended to be higher. A meta-analysis in 2008 calculated 3.8% [39]. A prospective study in 2005 reported 9.9% [20] and a retrospective study in 2010 4.9% [40]. While Fusco et al. report a prevalence rate of approximately 3 to 4% is higher than previously indicated [7, 41]. More recent retrospective studies of 2019 and 2022, however, estimated an incidence of 6.8% [36] or even 19.4% [21]. The reason for this tendency for incidences to be higher than in our work, is likely to be the longer period over which our data was collected and the fact that it was collected early. Also the definitions of MRONJ are in debate. The restrictive AAOMS criteria from 2014, which we used in this work, may underestimate the condition's true prevalence. By excluding cases that exhibit bone alterations without the possibility of bone probing through a fistula, the AAOMS guidelines risk overlooking significant clinical presentations. A broader definition could have provided a more accurate understanding MRONJ, capturing cases that would otherwise go unrecognized in numbers. Also MM patients could may be at a higher inherent risk for ONJ development [7]. Nowadays, oncologists and especially dentists and cranio-maxillo-facial surgeons are highly aware of the prevention and treatment of MRONJ. At our center, standardized dental evaluation began 2005. German and international guidelines recommend an initial dental screening before AR therapy starts and a close follow-up regimen during AR treatment [42]. These measures potentially increase the incidence and detection rates but might also improve the MRONJ outcomes [43].

In addressing the observed longer OS in MM patients with MRONJ, it is crucial to carefully consider certain limitations that may influence our interpretation of the results. Notably, our PSM did not account for co-morbidities, cardiovascular risk factors, renal function, or ECOG performance scores, all of which could significantly influence patient outcomes. The inability to achieve equal matching for the year of MM diagnosis and the exclusion of secondary malignancies from the PSM analysis might introduce bias into our findings. Furthermore, our study did not incorporate soft tissue involvement and autoSCT in the PSM process due to their status as follow-up or therapy markers, potentially limiting the scope of our analysis. Given the wide array of markers involved, we recognize that incorporating all of them into the PSM process may render it less feasible or even impractical. Regarding MRONJ, we included patients with onset dating back to 1993, despite the fact that the association between ONJ and AR medications was not formally described until 2003.The lack of information on the group of 'good' or 'better' responders to AR therapy, as well as the absence of tested or available surrogate markers, could impact the interpretation of our findings. Our study did not investigate the bone microenvironment, which could have provided deeper insights into the MRONJ disease process. Techniques such as osteodensitometry, further lab values or pathological bone evaluations might have contributed to a more comprehensive understanding of the underlying mechanisms; however, these approaches are not feasible in a retrospective study.

Further studies are needed to clarify the pathological process of MRONJ. Large prospective studies would make it possible to test how efficiently AR therapy reduces SRE. Such studies would also enable the examination of safety and important adverse events such as MRONJ. For further retrospective studies, additional disease-determining markers could be added to PSM. Included among these additional markers might be similar baseline bone findings, similar renal function, or similar soft tissue involvement of MM. It would also be useful to investigate AR therapy and other tumor types (e.g., breast cancer and prostate cancer) and examine OS depending on MRONJ development. OS and MRONJ incidence might be different in these other cancers. Further work may help to better identify the individual risk of MRONJ, the impact of bone microenvironment and improve survival and quality of life of tumor patients.

In conclusion, we were the first group to use NLP to handle large amounts of individual patient data in MM (*n* = 2389). We comprehensively analyzed a rare MRONJ adverse event. As a result, OS was 40 months longer in the matched comparison MRONJ group. We identified year of diagnosis and secondary malignancies as confounders. But this does not explain the large OS difference. On the contrary, the MRONJ group was slightly worse in several important clinical prognosis-determining details. Especially in ECOG Score 2 (12% vs. 6%), secondary malignancies (20% vs. 18%), year of diagnosis (2007 vs. 2012), cardiovascular risk factors (82% vs. 66%), less performed autoSCT (54% vs. 64%) and less used novel PI (62% vs. 86%) or IMiD (66% vs. 78%) drugs. One feasible explanation for survival benefit could be an altered bone microenvironment, which may also have contributed to the development of MRONJ. It is possible to hypothesize, that MRONJ patients do respond ‘better’ to AR medication and therefore develop more MRONJs, but may also live longer. This should be addressed in future translational projects.

### Supplementary Information

Below is the link to the electronic supplementary material.

Supplementary file1 (DOCX 36 kb)

Supplementary file2 (DOCX 37 kb)

Supplementary file3 (XLSX 9 kb)
