# Supplementary material for: Does medication-related osteonecrosis of the jaw affect survival of patients with Multiple Myeloma?: Exploring a large single center database using artificial intelligence
Source: Clin Exp Med. 2023 Oct 7;23(8):5215–26. doi: 10.1007/s10238-023-01100-6 (PMC10725344; doi:10.1007/s10238-023-01100-6)
Supplement: Supplementary file 2 — Supplementary file2 (DOCX 37 kb) [file 10238_2023_1100_MOESM2_ESM.docx]

Supplementary Material

# Supplementary Data- Results

*MM progression parameters*

We regard MM progression parameters as age, albumin, bone marrow infiltration, calcium level, extragradient, hemoglobin level, immunoglobulin heavy chain subtype, involved/uninvolved serum free light chain ratio, lactate dehydrogenase level, present bone lesions, present CAST nephropathy and cytogenetic (high) risk status, present extramedullary disease, renal function deterioration, serum free light chain kappa or lambda and β2-microglobulin. Other relevant parameters were smoking, drinking, obesity, cardiovascular diseases, and secondary malignancies. The differences between MRONJ and CTRL group are detailed in table 2 and 3. None of them are significantly different between the two groups.

**Table 6. MM progression risk laboratory results.**

|  |  | **MRONJ** | **CTRL** |  | **Total** | **Unit** |
| --- | --- | --- | --- | --- | --- | --- |
|  |  | *n (Range)* | *n (Range)* | *p-value* | *n (Range)* |  |
| **Albumin** |  | 3.9 (2.8 - 4.5) | 3.6 (2.2 - 4.8) | 0.091 | 3.7 (2.2 - 4.8) | *g/dl* |
| **β2-microglobulin** |  | 4.3 (1.4 - 17.6) | 5.6 (1.2 - 35.9) | 0.361 | 4.9 (1.2 - 35.9) | *mg/l* |
| **Bone marrow infiltration** |  | 47 (10 - 90) | 49 (10 - 90) | 0.726 | 48 (10 - 90) | *%* |
| **Extragradient** |  | 19 (0 - 61.2) | 29.3 (0 - 85.9) | 0.104 | 25 (0 - 85.9) | *g/l* |
| **Heavy chain** | IgA | 3591 (269 - 6830) | 2505 (299 - 6436) | 0.268 | 3125 (269 - 6830) | *mg/dl* |
|  | IgG | 4006 (1067 - 8320) | 4709 (1432 - 10138) | 0.286 | 4428 (1067 - 10138) | *mg/dl* |
| **Serum free light chains** | kappa | 1011 (5.4 - 10252) | 1834 (1.9 - 29280) | 0.519 | 1527 (1.9 - 29280) | *mg/l* |
|  | lambda | 795.7 (0.71 - 13000) | 387.8 (0.5 - 4718) | 0.533 | 541.9 (0.71 - 13000) | *mg/l* |
|  | K/L ratio | 262.7 (1.2 - 1239) | 486.2 (3.72 - 5248) | 0.478 | 389.5 (1.2 - 5248) |  |
| **Bence Jones proteinuria** | kappa | 543.2 (6.9 - 5150) | 419.1 (6.9 - 1590) | 0.756 | 472 (6.9 - 5150) | *mg/l* |
|  | lambda | 8.95 (3.9 - 14) | 920.4 (28 - 3930) | 0.417 | 717.9 (3.9 - 3930) | *mg/l* |
|  | K/L ratio | 139.3 (1.77 - 1321) | 107.5 (1.25 - 407.7) | 0.478 | 121.1 (1.25 - 1321) |  |
| **Blood calcium level** |  | 2.5 (1.9 - 4.34) | 2.47 (1.4 - 4.9) | 0.802 | 2.49 (1.4 - 4.9) | *mmol/l* |
| **Hemoglobin** |  | 11.1 (5.6 - 15.5) | 11.4 (6.9 - 17.7) | 0.669 | 11.2 (5.6 - 17.7) | *g/dl* |
| **LDH** |  | 203.1 (126 - 334) | 188 (81 - 435) | 0.401 | 194.5 (81 - 435) | *U/l* |

Abbreviations: LDH= Lactate dehydrogenase
